# RACIAL/ETHNIC GROUP DIFFERENCES IN REPORTED SELF-NEGLECT AMONG OLDER ADULTS

**DOI:** 10.1093/geroni/igae098.1825

**Published:** 2024-12-31

**Authors:** Kenneth Steinman, Jim Pellerin, Yang Shi

**Affiliations:** The Ohio State University, Columbus, Ohio, United States; The Ohio State University, Columbus, Ohio, United States; The Ohio State University, Columbus, Ohio, United States

## Abstract

Self-neglect is a pernicious problem and represents the most common type of report investigated by adult protective services (APS). Calls for culturally competent approaches to addressing self-neglect in older adults have been undermined by the lack of research on racial/ethnic group differences and how they may vary across communities. Analyses of 101,015 reports of self-neglect among people 65+ years old validated by the Texas APS system from 2021 through 2023 found the incidence was nearly twice as high for (non-Hispanic) African Americans as for other older adults (IRR=1.91, 95%CI: 1.88, 1.94), whereas the difference for Hispanics was modest (IRR=0.95, 95%CI: 0.93, 0.96). Stratified analyses, however, found marked variation across the state’s 11 regions. In rural regions of West Texas, the higher reporting rates for African Americans were especially pronounced: three to four times the rates for other older adults. And in rural regions of East Texas, reporting rates for Hispanics were only one half to one third those of other older adults. Less variation was observed across major metropolitan regions. Further analyses assessed how regional population demographics and the racial/ethnic composition of APS staff may explain these divergent results. Efforts to understand and address racial/ethnic group differences in self-neglect should reconsider the propriety of describing groups as “underserved” when they may be disproportionately more likely to be investigated by APS. When addressing self-neglect in a diverse client population, agencies should consider the background of staff and whether distinct approaches for are needed in rural areas.

